# Lead Poison

**Published:** 1882-07

**Authors:** 


### LEAD POISON.

All who use water, conducted into their dwellings by leaden pipes, are interested in the question, whether the lead, under any circumstances, can impregnate the water with poison? It is certainly so. No argument, beyond that of often ascertained facts, is necessary to prove this. But the water delivered to one family will cause a slow, wasting, and fatal disease, while the water from the same sources, introduced into the next house, is used for years without any appreciable ill results. The simple reason is, that in the fatal case, *the flow of water is obstructed*, either by a too sudden bend in the pipe, or by a pebble or other indestructible impediment. *Standing water will corrode lead.* Simple dampness will corrode lead, and bring out its poisonous qualities. Obstructions to a flowing stream of water will arrest any particles in that water which are not the pure water itself: those particles are usually of vegetable origin; and as soon as a small portion of them are collected in any part of the pipe, or rather arrested by the obstacle, destructive decomposition begins; and the gases escaping in consequence of this process act upon the lead, and make it poisonous. Every man, therefore, who builds a home for himself, should understand that, if it is to be supplied with water by leaden pipes, his life, and that of all his, depends on the fidelity of the plumber; and as plumbers trust their work to apprentice boys, and uninterested journeymen, the owner should watch the laying of every foot of pipe, and not allow an inch of it to be put down during his absence; the points to which he should bend his most fixed attention are:

*First.* Let the pipe be laid as straight as possible.

*Second.* Let every joint be made perfectly smooth.

*Third.* See to it, that not an atom of anything be left inside the pipe which would obstruct the smallest particle of any substance, whether it be leaf, or wood, or grass, or hair, or string, or worm, or insect, or anything else.

Meanwhile, as long as it is certain that still water corrodes lead, the most unthinking person will draw the practical inference, that the water from the hydrant should be allowed to run off for the first five or ten seconds after turning the faucet, to get a supply for drinking or eating.

